# A Durable and Self-Cleaning Superhydrophobic Surface Prepared by Precipitating Flower-Like Crystals on a Glass-Ceramic Surface

**DOI:** 10.3390/ma13071642

**Published:** 2020-04-02

**Authors:** Haiqing Fu, Shuo Liu, Lanlin Yi, Hong Jiang, Changjiu Li, Yongjun Chen

**Affiliations:** 1 Special Glass Key Lab of Hainan Province, Haikou 570228, China; fuhaiqing18@sina.com (H.F.); lshuo1@outlook.com (S.L.); yilanlin20@gmail.com (L.Y.);; 2 State Key Laboratory of Marine Resource Utilization in South China Sea, Hainan University, Haikou 570228, China; 3 College of Materials Science and Engineering, Hainan University, Haikou 570228, China

**Keywords:** glass-ceramic, crystallization, dual-scale roughness, superhydrophobic, durable, self-cleaning

## Abstract

Herein, a superhydrophobic surface with superior durability was fabricated on a glass-ceramic surface by crystallization, hydrofluoric acid (HF) etching, and surface grafting. The as-prepared glass-ceramic surface was composed of three-dimensional flower-like micro-clusters, which were self-assembled from numerous nanosheets. Such a dual-scale rough surface exhibited superhydrophobicity, with a water contact angle (WCA) of 170.3° ± 0.1° and a sliding angle (SA) of ~2° after grafting with 1H, 1H, 2H, 2H-perfluorodecyltriethoxysilane (FAS-17). This can be attributed to the synergistic effect between the dual-scale structure and surface chemistry. Furthermore, this surface exhibited excellent self-cleaning properties, stability against strong acid and strong alkali corrosion, and anti-stripping properties.

## 1. Introduction 

**Figure 1:**
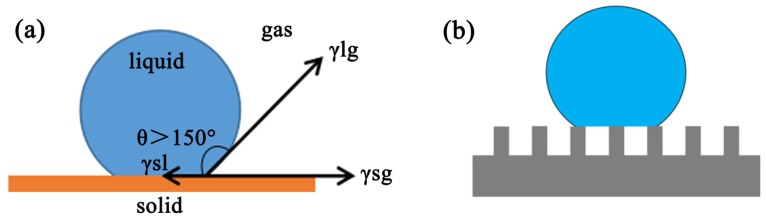
(**a**) Schematic of a superhydrophobic surface, (**b**) Cassie–Baxter model.

Inspired by the “lotus effect” in nature, numerous biomimetic superhydrophobic surfaces have been widely studied [1,2,3,4]. Superhydrophobicity is an extreme wetting phenomenon, which can be distinguished by measuring the contact angle (CA). The CA is the angle (*θ*) between the solid-liquid interface and the gas-liquid interface tangent at the solid-liquid-gas three-phase interface (Figure 1a). A value of *θ* is greater than 150° corresponds to superhydrophobicity [5,6]. Superhydrophobic surfaces have a wide range of application prospects for self-cleaning [7,8,9], anti-icing [10], anti-corrosion [9,10], oil-water separation [11,12], anti-fogging [13,14], and drag reduction [15,16].

Low surface energy and micro-nanostructures of a rough surface are the main determinants of superhydrophobicity [17]. Therefore, a superhydrophobic surface can be obtained by either (1) modification of a rough substrate via low surface energy or (2) fabrication of a rough surface on a low-surface-energy substrate [18]. Currently, superhydrophobic surfaces are fabricated by several preparation techniques, such as sol-gel [19], templating [20], chemical vapor deposition (CVD) [21], electrospinning [21,22], laser/plasma etching [7,23,24], and phase separation [25]. For instance, Xue et al. [19] constructed a dual-scale surface roughness by coating fibers with titania sol and demonstrated an optimal superhydrophobicity with a water contact angle (WCA) of 154.0° ± 0.5°. Ma et al. [21] proposed a combination of electrospinning and CVD to prepare superhydrophobic fabrics with a WCA of 175° and a sliding angle (SA) <2.5°. Wang et al. [23] utilized picosecond laser pulses and fabricated a superhydrophobic solar glass by constructing a groove-shaped array on the glass surface, obtaining a CA of 156°. Gao et al. [24] designed a superhydrophobic surface by fabricating leaf-like clusters on a zinc surface by the plasma etching technique and demonstrated a WCA of 158° and an SA <5°.

It is well known that glass-ceramic exhibits high mechanical strength, excellent corrosion resistance, desirable abrasive resistance, and high thermal stability [26,27,28]. Recently, our group has successfully prepared a superhydrophobic glass-ceramic surface, with an irregular porous coral-like structure, which demonstrated a WCA of 152° and an SA of <5° [29]. However, the size and distribution of the crystals were found to be different on the surface. The surface roughness and fine structure were not well controlled. Based on this, we used Cr_2_O_3_ as the crystal-nucleating agent and nepheline as the basic microcrystalline system to prepare a superhydrophobic glass-ceramic surface with controllable and finer three-dimensional structure. 

Herein, we choose Cr_2_O_3_ as a nucleating agent, which can form octahedral crystal nucleus. Then, nepheline crystals were grown vertically on the outer surface of the octahedron to form a flower-like structure. The superhydrophobic glass-ceramic surfaces were prepared in two steps. In the first step, a flower-like structure was precipitated on the glass-ceramic surface by heat treatment, which was subsequently treated with hydrofluoric acid (HF) to corrode the glass phase and obtain a certain roughness by exposing the crystals. In the second step, the glass-ceramic surface was grafted with 1H, 1H, 2H, 2H-perfluorodecyltriethoxysilane (FAS-17) to obtain a superhydrophobic surface. The results revealed that the as-prepared glass-ceramic surface possesses a dual-scale hierarchical microstructure and exhibits a WCA of 170.3° ± 0.1° and a SA of ~2° after surface grafting. Moreover, the surface exhibits excellent self-cleaning properties, stability against strong acid and strong alkali corrosion, and anti-stripping properties. Furthermore, the mechanism of superhydrophobic self-cleaning behavior was explained based on the Cassie-Baxter model (Figure 1b). In this model, the grooves absorb a large amount of air, which reduces the solid-liquid contact area and effectively prevents water droplets from penetrating into the grooves.

## 2. Experimental

### 2.1. Materials 

In this study, a Na_2_O-Al_2_O_3_-SiO_2_ based glass-ceramic system was investigated. The raw materials, including sodium carbonate (Na_2_CO_3_ ≥ 99.5%), magnesium oxide (MgO ≥ 98.0%), aluminum oxide (Al_2_O_3_ ≥ 99.0%), silica (SiO_2_ ≥ 99.0%), potassium carbonate (K_2_CO_3_ ≥ 99.0%), ferric oxide (Fe_2_O_3_ ≥ 99.0%), chromic oxide (Cr_2_O_3_ ≥ 99.0%), and FAS-17 (≥96.0%) were purchased from Macklin Biochemical Co., Ltd., Shanghai, China. Calcium carbonate (CaCO_3_ ≥ 99.0%), boric acid (H_3_BO_3_ ≥ 99.5%), HF (40.0%), and ethanol (C_2_H_5_OH ≥99.8%) were obtained from Xilong Chemical Co., Ltd., Guangzhou, China. 

### 2.2. Fabrication of a Superhydrophobic Glass-Ceramic Surface 

Table 1 presents the chemical composition of the investigated glass-ceramic. The completely mixed reagent powders were melted in an alumina crucible at 1350 °C for 2 h to obtain a homogeneous molten glass. Then, the melt was poured into water to obtain a glass frit, followed by crushing and milling. A cylindrical glass sample was obtained by pressing the glass powder under a uniaxial pressure of 5 MPa and then was placed on an alumina substrate for subsequent thermal treatment.

**Figure 2:**
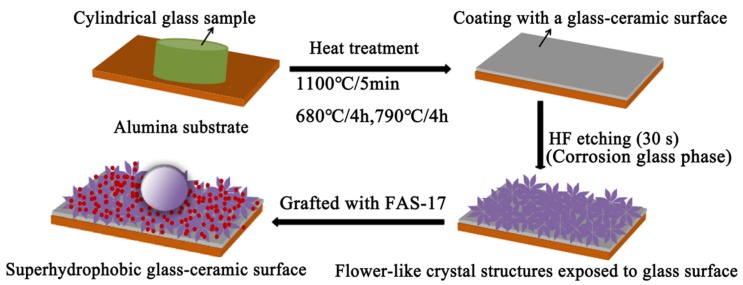
Schematic illustration of the preparation of a durable superhydrophobic glass-ceramic surface.

The heat-treated glass-ceramic surface was etched in a 5 vol.% HF solution for 30 s to expose the crystals. Then, the HF-etched samples were immersed into a 2 wt.% ethanol solution of FAS-17 at 60 °C for 2 h and dried in an oven for 2 h at 120 °C to obtain fluorinated surfaces [23,29]. Figure 2 illustrates the preparation of a durable superhydrophobic glass-ceramic surface.

### 2.3. Material Characterization 

Glass transition temperature (*T_g_*), crystallization temperature (*T_c_*), and melting point (*T_m_*) were measured by differential thermal analysis (DTA, NETZSCH STA449F5, Selb, Germany) in a nitrogen atmosphere. The DTA curve was obtained by heating in the range from 40 to 1250 °C at a heating rate of 10 °C min^−1^. The crystalline phase of the glass-ceramic was determined by X-ray diffraction (XRD, Bruker D8 Advance X, Aachen, Germany) with CuKα radiation. The XRD pattern was obtained in the diffraction angles (2θ) range of 15 to 75°, with a step size of 0.02°. The microstructure of the glass-ceramic was characterized by a field emission scanning electron microscope (FESEM, Hitachi S-4800, Tokyo, Japan) equipped with an energy-dispersive spectrometry (EDS) system. The three-dimensional structure was measured by a laser scanning confocal microscope (VK-X250K, Keyence, Osaka, Japan). The chemical composition was analyzed by EDS and X-ray photoelectron spectroscopy (XPS, ESCALAB 250Xi, Waltham, MA, USA). The WCA and SA were measured by using an optical contact angle meter (Drop meter A-100, Ningbo, China). Each surface was measured at five different points with a water droplet of ~5 μL. 

## 3. Results and Discussion

### 3.1. Crystallization Behavior 

**Figure 3:**
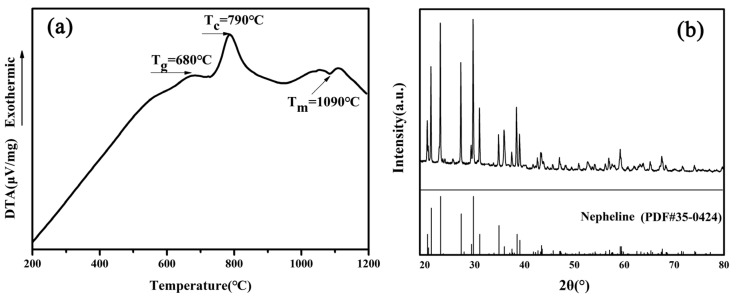
(**a**) DTA curve of the parent glass and (**b**) XRD pattern of a glass-ceramic sample nucleated at 680 °C for 4 h and crystallized at 790 °C for 4 h. (*T_g_*): transition temperature, (*T_c_*): crystallization temperature, (*T_m_*): melting point.

Figure 3a presents the DTA curve of the parent glass, showing an exothermic crystallization peak at 790 °C. The DTA curve indicates that the *T_g_* and *T_m_* were ~680 °C and ~1090 °C, respectively. Based on the DTA results, the nucleation and crystallization temperature of glass-ceramic were 680 °C and 790 °C, respectively. Figure 3b shows the XRD pattern of a glass-ceramic sample nucleated at 680 °C for 4 h and crystallized at 790 °C for 4 h, confirming the presence of the nepheline phase (PDF#35-0424).

### 3.2. Analysis of Surface Morphology

**Figure 4:**
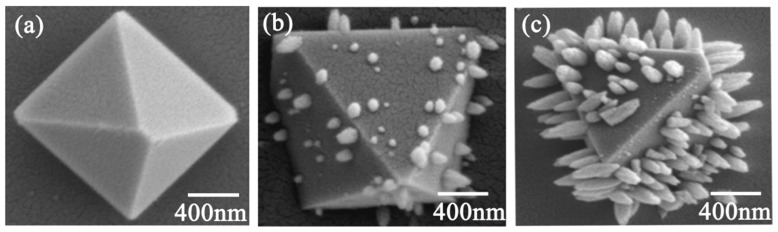
SEM images of samples nucleated at 680 °C for (**a**) 2 h, (**b**) 4 h, and (**c**) 12 h.

Figure 4 shows the samples nucleated at 680 °C for 2 h, 4 h, and 12 h. The crystal nuclei of the octahedra were precipitated after nucleation for 2 h, and the main components were Cr and O (Figure 4a and Figure S1). The nepheline crystals began to grow vertically on the outer surface of the octahedron after nucleation for 4 h (Figure 4b). The length of the nepheline crystal reached 400 nm after nucleation for 12 h (Figure 4c). The EDS pattern indicated that the vertically grown crystals were nepheline, because their main components were Na, Al, and Si, as for nepheline (Figure S2).

**Figure 5:**
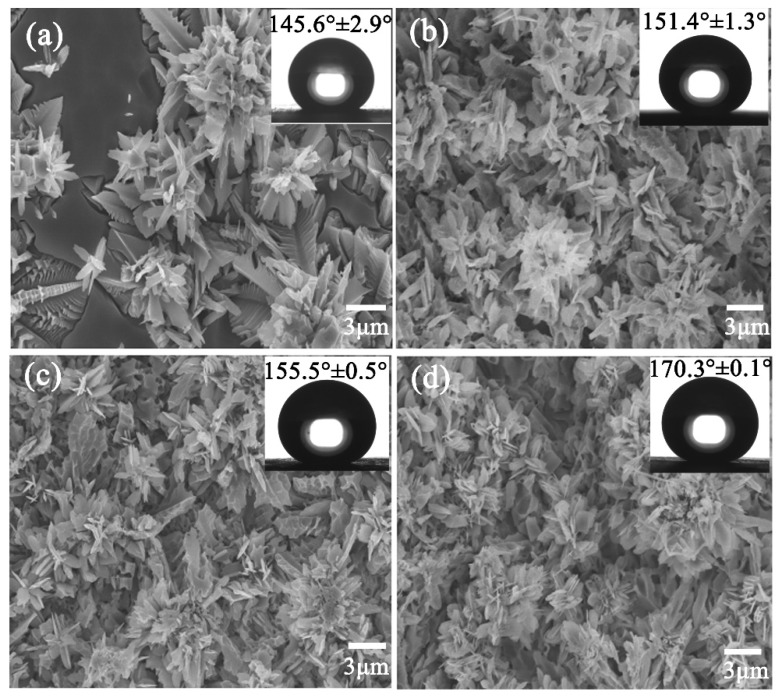
SEM images of the samples nucleated at 680 °C for 4 h and crystallized at 790 °C for (**a**) 0.5 h, (**b**) 1 h, (**c**) 2 h, and (**d**) 4 h. The inset shows the water contact angle (WCA) values for different crystallization times.

After nucleation at 680 °C for 4 h, the nepheline crystals began to precipitate on the outer surface of the octahedral nuclei. (Figure 4b). Therefore, we established a heat treatment system to observe the crystal growth process by nucleation at 680 °C for 4 h and crystallization at 790 °C for different times. Before SEM observation, the glass phase was corroded with an HF solution (5 vol.%) for 30 s to expose the nepheline crystals. Figure 5 exhibits SEM images of the glass-ceramic surface. It can be readily observed that the micro-clusters, with a special flower-like structure, were composed of numerous sheet-like nanocrystals. Moreover, variation in crystal growth was observed by varying the crystallization time. For instance, after the crystallization for 0.5 h (Figure 5a), a large number of microplates self-assembled into several micro-flowers. Once the crystallization time reached 1 h, numerous sheet-like nanocrystals and micro-flowers were observed on the glass-ceramic surface (Figure 5b). A further increase in crystallization time led to an increase in the surface density of micro-clusters and nanosheets (Figure 5c). When the crystallization time was close to 4 h, large and continuous flower-like micro-clusters, covering the entire surface of the glass-ceramic, were observed (Figure 5d). After HF etching and FAS-17 grafting, the WCA values of the sample surfaces crystallized for 0.5, 1, 2, and 4 h were 145.6° ± 2.9°, 151.4° ± 1.3°, 155.5° ± 0.5°, and 170.3° ± 0.1°, respectively (Figure 5), and the root-mean-square surface roughness (RMS) of the samples after crystallization for 0.5 h, 1 h, 2 h, and 4 h were 1.746 μm, 2.196 μm, 2.357 μm, and 2.810 μm, respectively (Figure S3). As the crystallization time increased, the flower clusters became larger and larger, leading to an increase in surface roughness. Therefore, the WCA also increased with the increase of crystallization time after HF etching and FAS-17 grafting. Noteworthy, grafting with FAS-17 only reduced the surface energy of glass-ceramic and did not change its structure.

**Figure 6:**
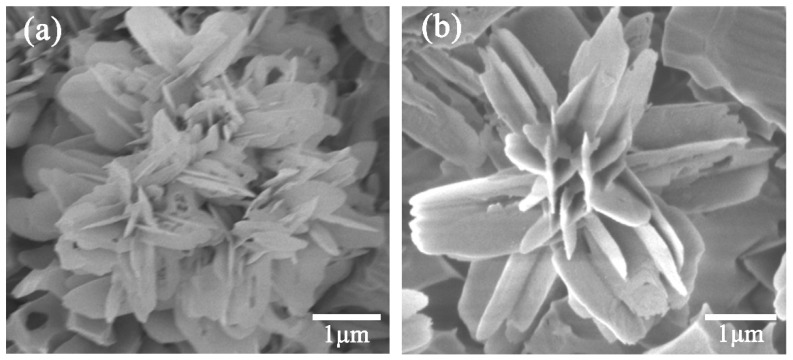
SEM images of (**a**) a single flower-like micro-cluster and (**b**) a single flower-like structure after nucleation at 680 °C for 4 h and crystallization at 790 °C for 4 h.

Figure 6 presents SEM images of a single flower-like micro-cluster and a single flower-like structure. Overall, the morphology of glass-ceramic surface was characterized by flower-like micro-clusters, with lateral dimension of 2 to 15 μm, as shown in Figure 5d and Figure 6a. These micro-clusters self-assembled from a large number of flower-like structures. Moreover, numerous nanosheets self-assembled to form flower-like structure with a length of 0.1–2 μm and width of 50–900 nm (Figure 6b). These results clearly show that the glass-ceramic surface had a dual-scale hierarchical structure, where the first layer consisted of micron-scale protrusions, and the second layer contained nano-scale features. It is worth emphasizing that the observed dual-scale microstructure was able to capture air and F groups, which resulted in high WCA.

### 3.3. Surface Wettability and Self-Cleaning Properties

**Figure 7:**
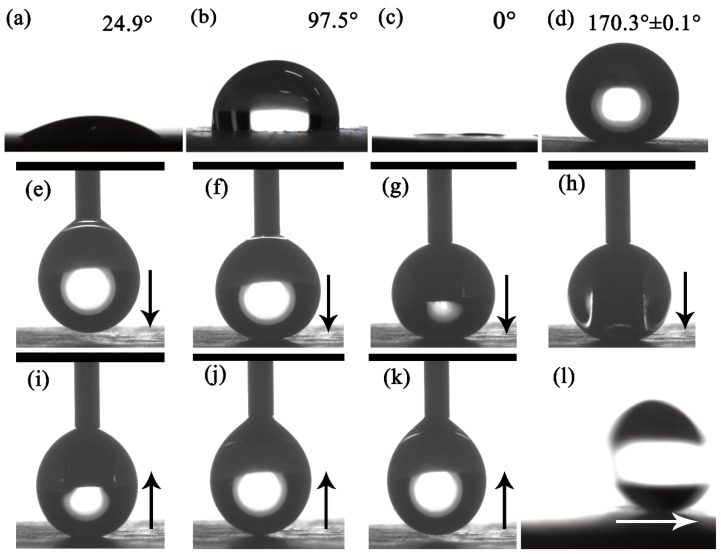
Optical images of the WCA on glass-ceramic surfaces: (**a**) original glass-ceramic surface; (**b**) 1H, 1H, 2H, 2H-perfluorodecyltriethoxysilane (FAS-17)-grafted original glass-ceramic surface; (**c**) hydrofluoric acid (HF)-etched glass-ceramic surface; and (**d**) HF-etched and FAS-17-grafted glass-ceramic surface; (**e**–**k**) process of water droplet approach, contact, deformation, and detachment for HF-etched and FAS-17-grafted glass-ceramic surfaces. The arrow represents the direction of movement of the needle; (**l**) a water droplet sliding on the surface.

Figure 7a–d presents the WCA of original and HF-etched glass-ceramic surfaces. The WCA of the original glass-ceramic surface was 24.9°, indicating the hydrophilic nature of the original surface (Figure 7a). However, the WCA of the FAS-17-grafted original glass-ceramic surface increased to 97.5° (Figure 7b), which indicates that roughness plays a critical role in superhydrophobicity. On the other hand, the HF-etched glass-ceramic surface exhibited superhydrophilicity, with a WCA of 0° (Figure 7c). Compared to the original glass-ceramic surface, the exposed crystals of the HF-etched glass-ceramic surface increased the surface roughness and provided superhydrophilicity [30]. In contrast, when the HF-etched glass-ceramic surface was grafted with FAS-17, a WCA of 170.3° ± 0.1° was achieved due to low surface energy and dual-scale roughness (Figure 7d). Therefore, it can be concluded that the superhydrophobic surface was the result of a combination of surface roughness and low surface energy. Herein, the surfaces were nucleated at 680 °C for 4 h and crystallized at 790 °C for 4 h, then etched in an HF solution (5 vol.%) for 30 s. Figure 7e–k presents the process of water droplet approach, contact, deformation, and detachment for the HF-etched and FAS-17-grafted glass-ceramic surfaces. The water droplet retained its typical spherical shape after contacting the glass-ceramic surface and easily separated from the surface. Figure 7l shows the sliding process of the water droplet (7 μL) on the surface, demonstrating the superhydrophobicity of the surface, with a low SA of ~2°. Obviously, the as-prepared glass-ceramic surface showed low adhesion.

**Figure 8:**
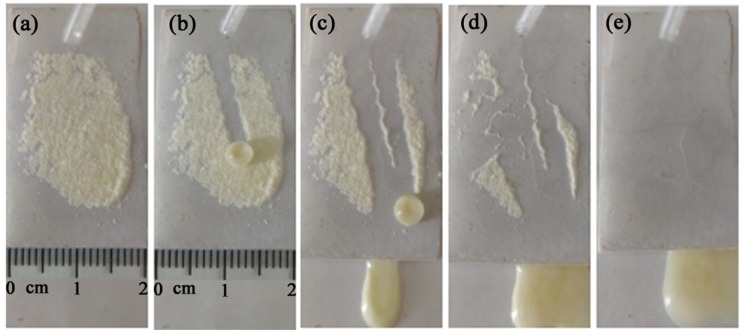
(**a**–**e**) Self-cleaning process of the superhydrophobic glass-ceramic surface.

Figure 8 shows the process of self-cleaning of the superhydrophobic glass-ceramic surface. Glass powder was used as a model pollutant. A layer of pollutant was sprinkled on the superhydrophobic glass-ceramic surface tilted at an angle of ~5°. When distilled water droplets (~50 μL) came in contact with the contaminated surface, the pollutant became attached to the water droplets and was removed (Figure 8a–d), resulting in a cleaned glass-ceramic surface (Figure 8e). Moreover, the water droplet maintained its spherical shape even after absorbing the pollutants. This result clearly revealed the self-cleaning behavior of the superhydrophobic glass-ceramic surface.

For the comprehensive understanding of the self-cleaning performance of the superhydrophobic sample surface with a flower-like microstructure, we described the WCA using the Cassie–Baxter Equation as follows [31]:

(1) $$\mathrm{Cos}\;\theta_{A}=f_{1}\mathrm{Cos}\;\theta -f_{2}$$

where *θ*_A_ (170.3°) and *θ* (97.5°) represent the WCA of the flower-like and the original glass-ceramic surfaces grafted with FAS-17, respectively; and *f*_1_ and *f*_2_ refer to the fractional area of the flower-like structure and that of the air between the structural voids, respectively (i.e., *f*_1_ + *f*_2_ = 1). Equation (1) shows that the WCA of the flower-like surface (*θ*_A_) increased with an increasing fraction of air (*f*_2_). Based on Equation (1), the *f*_2_ value of the flower-like surface was found to be 0.9835, indicating that air accounted for ~98.35% of the contact area between the flower-like structure and the water droplets. This result shows that the dual-scale structure plays a critical role in the superhydrophobicity of glass-ceramic surfaces. Moreover, the presence of a dual-scale microstructure also explains the excellent self-cleaning performance of glass-ceramic surfaces.

**Figure 9:**
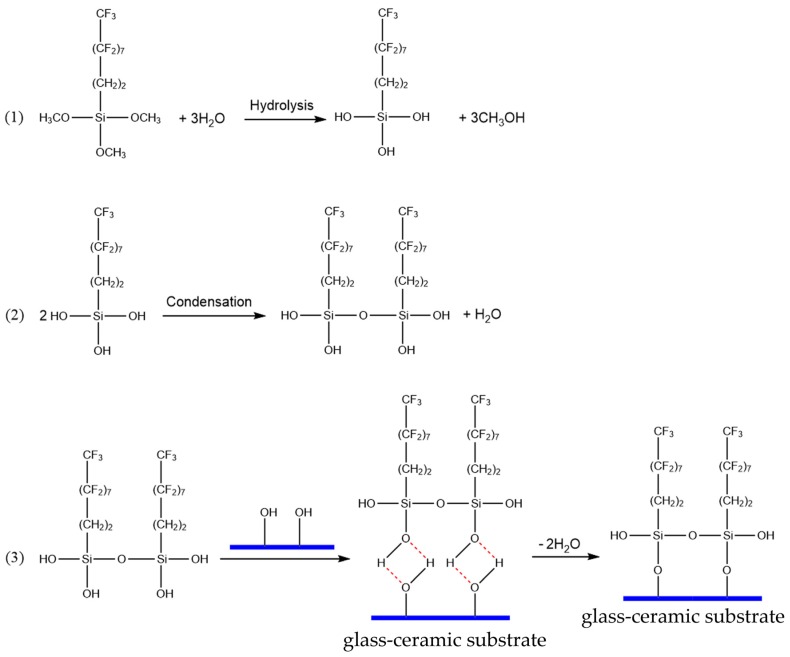
The reaction process between FAS-17 and glass-ceramic.

Equations (2) and (3) show the mechanism of hydrolysis and condensation of compounds, respectively [32,33,34,35]. The reaction process between FAS-17 and the glass-ceramic substrate involves hydrolysis and condensation and can be divided into three steps (Figure 9). In the first step, the fluoroalkylsilane is hydrolyzed to form siloxanes. In the second step, the siloxanes are condensed to form oligosiloxanes. Finally, the Si–OH in the oligomers form hydrogen bonds with the OH groups on the glass-ceramic substrate and covalent bonds with the glass-ceramic substrate during drying and curing [36]. As a result, low-surface-energy groups were successfully grafted onto the glass-ceramic.

(2) $$\mathrm{Hydrolysis}:\;M{\left(OR\right)}_{n}+nH_{2}O\to M{\left(OH\right)}_{n}+nROH$$

(3) $$\mathrm{Condensation}:\;M{\left(OH\right)}_{n}\to MO_{n/2}+n/2H_{2}O$$

### 3.4. Surface Chemical Composition

Furthermore, EDS was employed to analyze the composition of the glass-ceramic surface. The as-prepared sample surface mainly contained O, Cr, Na, Al, and Si, as shown in Figure S4. However, the FAS-17-grafted glass-ceramic surface exhibited the presence of F as well, which indicated the successful implantation of FAS-17 on the glass-ceramic.

**Figure 10:**
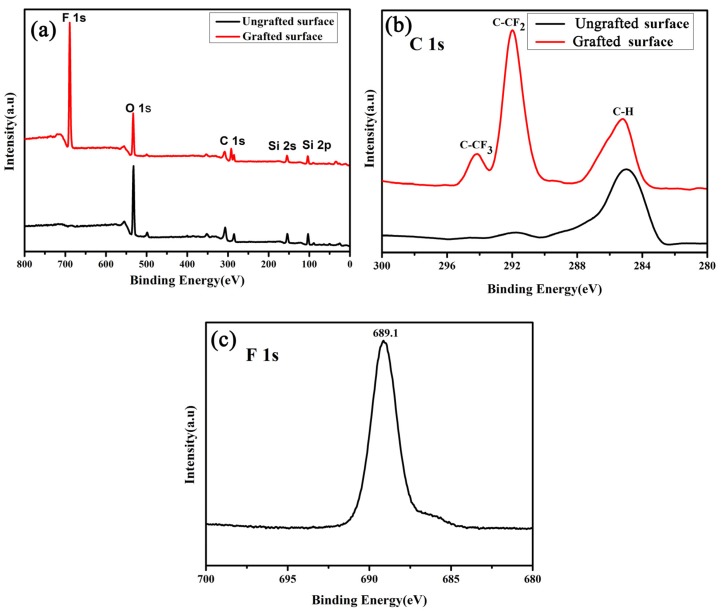
(**a**) Wide-range, (**b**) high-resolution C 1s and (**c**) F 1s XPS spectra of the glass-ceramic surfaces.

The state of the F element was further analyzed by XPS (Figure 10a–c). The as-prepared glass-ceramic surface mainly contained O 1s, C 1s, Si 2s, and Si 2p peaks (Figure 10a), which were located at binding energies of 532.6, 284.7, 155.0, and 103.2 eV, respectively. The FAS-17-grafted glass-ceramic surface contained an additional peak of F 1s at 689.1 eV (Figure 10a,c), indicating the presence of F groups on the grafted glass-ceramic surface. Figure 10b presents high-resolution C 1s spectra of the glass-ceramic surfaces. The C 1s spectrum of the FAS-17-grafted glass-ceramic surface showed three peaks, located at the binding energies of 285.2, 291.3, and 294.1 eV, which can be ascribed to C–H, C–CF_2_, and C–CF_3_ bonds [23,37], respectively. It is well known that, among these three groups, the surface energy of C–CF_3_ is the lowest (6 mN m^−1^), followed by that of C–CF_2_ [38]. The presence of the C–CF_3_ and C–CF_2_ groups reduced the surface energy, leading superhydrophobicity. XPS analysis further confirmed the successful grafting of FAS-17 on the sample.

### 3.5. Surface Durability Test

**Figure 11:**
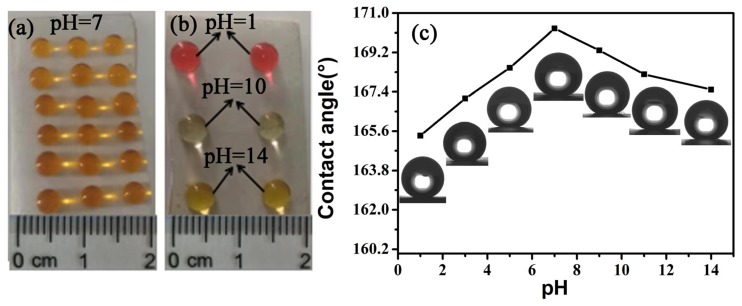
(**a**,**b**) Digital photographs of water droplets with different pH on the superhydrophobic glass-ceramic surface and (**c**) changes of WCA after the samples were immersed in H_2_SO_4_ and NaOH solutions (pH = 1, 3, 5, 7, 9, 11, 14) for 48 h.

Durability of a superhydrophobic surface is indispensable for its practical applications. Therefore, several tests were carried out to evaluate the durability of the glass-ceramic surface. In practical applications, a superhydrophobic surface may come into contact with strong acids or bases. The FAS-17-grafted glass-ceramic surface exhibited superior stability against aqueous H_2_SO_4_ (pH = 1) and aqueous NaOH (pH = 14) (Figure 11). Figure 11 shows digital photographs of water droplets with different pH on the superhydrophobic glass-ceramic surface. Distilled water was dyed with methyl orange to determine the pH of the solution based on the color change. These droplets showed a spherical shape on the glass-ceramic surface. The as-prepared superhydrophobic glass-ceramic surface appeared uniform (Figure 11a) and resistant to the attack of the strong acid and alkali (Figure 11b). Herein, the volume of each water droplet was ~50 μL. Figure 11c presents the changes of WCA after the samples were immersed in H_2_SO_4_ and NaOH solutions (pH = 1, 3, 5, 7, 9, 11, 14) for 48 h, showing that the WCA remained above 165° for the different pH of the water droplets. 

**Figure 12:**
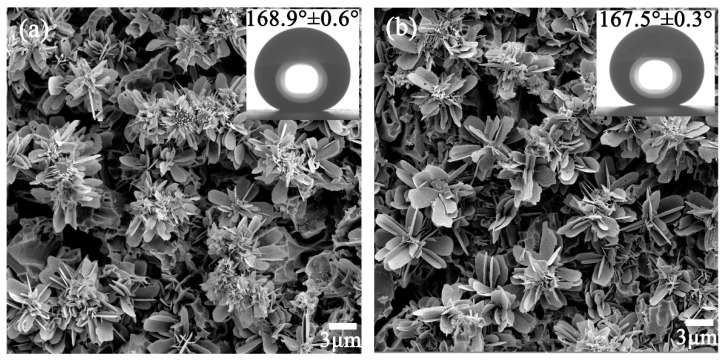
SEM images after peeling for (**a**) 10 and (**b**) 20 times with 3M scotch tape. Inset: WCA values.

Furthermore, the durability of the superhydrophobic glass-ceramic surface was demonstrated by the tape peeling test. After peeling for 10 and 20 times with 3M scotch tape (cat. 600), the glass-ceramic surfaces still maintained their special flower-like structure, and the WCA remained higher than 167° (Figure 12). 

## 4. Conclusions

In summary, a superhydrophobic glass-ceramic surface was fabricated by sequential crystallization, HF etching, and FAS-17 grafting. The as-prepared glass-ceramic surface was found to be composed of complex flower-like micro-clusters, which self-assembled from numerous nanosheets, showing a dual-scale hierarchical structure at the micro-/nanoscale. This dual-scale hierarchical structure is advantageous for capturing air to decrease the solid–liquid interface and for adsorbing more F groups to decrease the surface energy. The FAS-17-grafted glass-ceramic surface exhibited superior superhydrophobicity, with a WCA of 170.3° ± 0.1° and a SA of ~2°. Moreover, excellent self-cleaning performance and durability were also observed. The excellent properties of superhydrophobic glass-ceramic surfaces demonstrate the potential of glass-ceramic materials for a wide array of industrial applications, such as the fabrication of superhydrophobic glass-ceramic glazes for ceramic tiles and building materials.

## Figures and Tables

**Table 1:** Chemical composition of the studied glass-ceramic.

Oxide	SiO_2_	Al_2_O_3_	CaO	MgO	K_2_O	Fe_2_O_3_	Na_2_O	B_2_O_3_	Cr_2_O_3_
Amount (mol.%)	50.31	15.58	2.05	3.8	2.08	0.24	19.91	5.12	0.91

## Supplementary Materials

The following are available online at https://www.mdpi.com/1996-1944/13/7/1642/s1; Figure S1. (a) The sample was nucleated at 680 °C for 2 h, (b) EDS pattern marked with square in (a); Figure S2. (a) The sample was nucleated at 680 °C for 12 h, (b) EDS pattern marked with square in (a); Figure S3. Three-dimensional images of the samples nucleated at 680 °C for 4 h and crystallized at 790 °C for (a) 0.5 h, (b) 1 h, (c) 2 h, and (d) 4 h, where (e), (f), (g), and (h) are the high-magnification (150X) three-dimensional images of the regions in (a), (b) (c), and (d), respectively; Figure S4. EDS patterns of ungrafted and FAS-17-grafted glass-ceramic surfaces.
