# Supplementary material for: A Durable and Self-Cleaning Superhydrophobic Surface Prepared by Precipitating Flower-Like Crystals on a Glass-Ceramic Surface
Source: Materials (Basel). 2020 Apr 2;13(7):1642. doi: 10.3390/ma13071642 (PMC7178295; doi:10.3390/ma13071642)
Supplement: Supplementary file 1 [file materials-13-01642-s001.zip › Supplementary/materials-740600-supplementary-final.docx]

Supplementary materials

A Durable and Self‑Cleaning Superhydrophobic Surface Prepared by Precipitating Flower‑like Crystals on a Glass‑Ceramic Surface

Haiqing Fu ^1,2,3^, Shuo Liu ^1,3^, Lanlin Yi ^1,2,3^, Hong Jiang ^1,2,3^, Changjiu Li ^1,2,3,^* and Yongjun Chen ^2,3^

^1^ Special Glass Key Lab of Hainan Province, 570228 Haikou, China; [fuhaiqing18@sina.com](javascript:void(0);) (H.F.); [lshuo1@outlook.com](javascript:void(0);) (S.L.); [yilanlin20@gmail.com](javascript:void(0);) (L.Y.); [jianghong@hainanu.edu.cn](javascript:void(0);) (H.J.)

^2^ State Key Laboratory of Marine Resource Utilization in South China Sea, Hainan University, 570228 Haikou, China; [yongchen@hainanu.edu.cn](javascript:void(0);)

^3^ College of Materials Science and Engineering, Hainan University, 570228 Haikou, China.

***** Correspondence: lichangjiu@hainanu.edu.cn; Tel: +86‑186‑8958‑9458


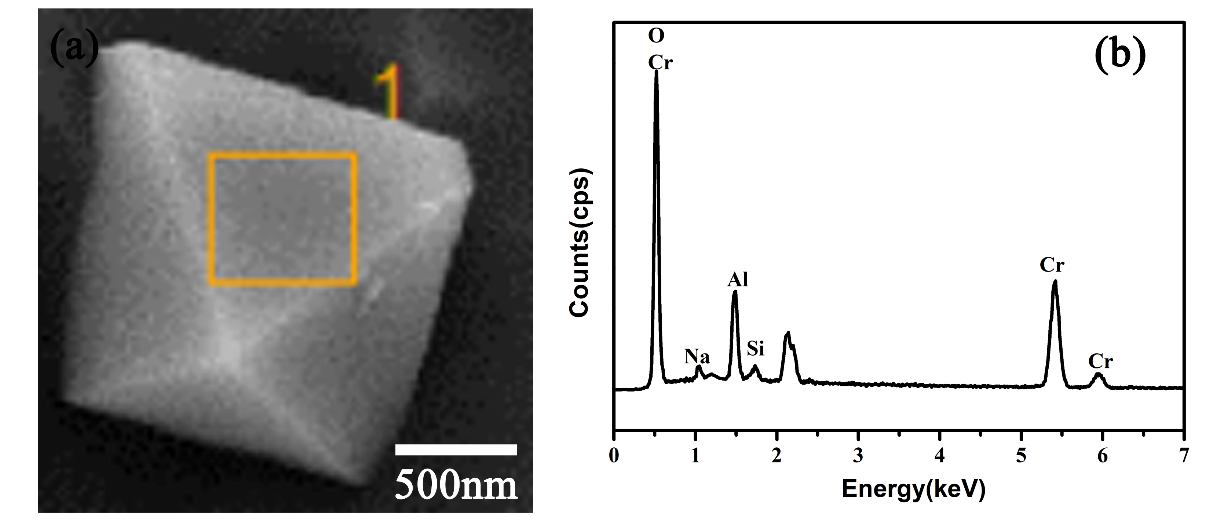


**Figure S1.** (**a**) The sample was nucleated at 680 °C for 2 h, (**b**) EDS pattern marked with square in (**a**).


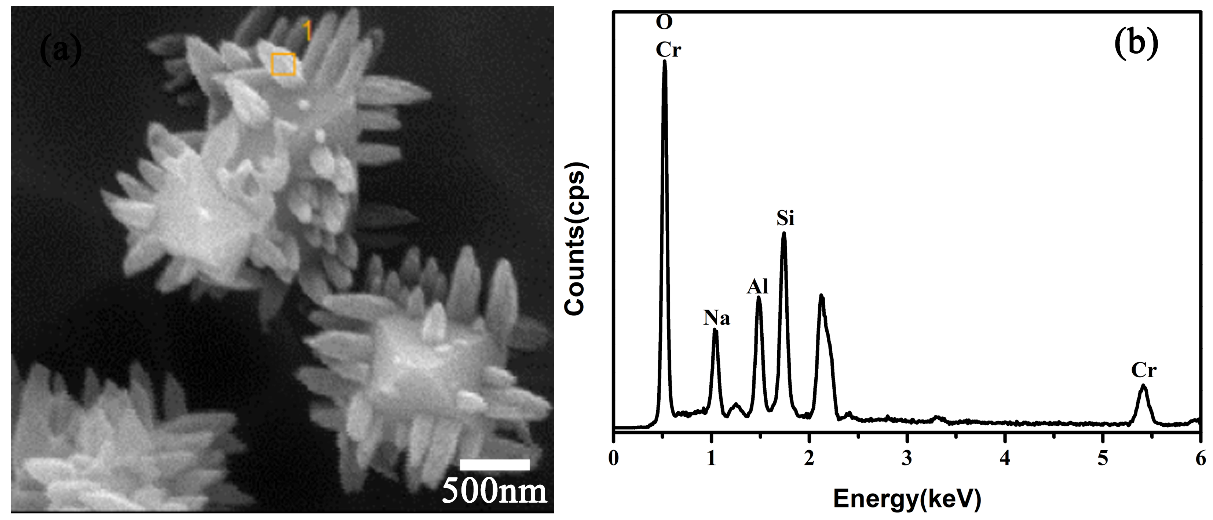


**Figure S2.** (**a**) The sample was nucleated at 680 °C for 12 h, (**b**) EDS pattern marked with square in (**a**).


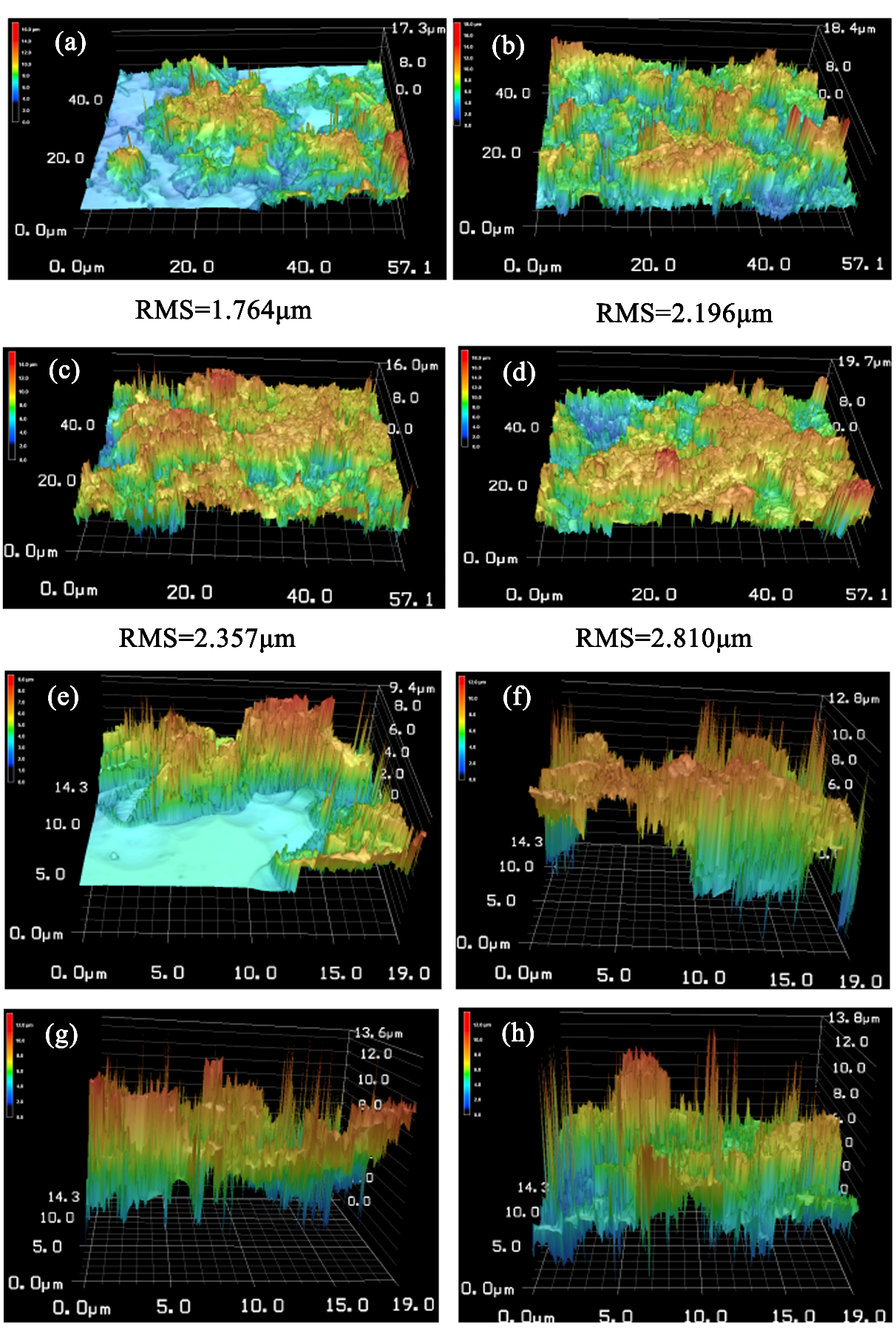


**Figure S3.** Three‑dimensional images of the samples nucleated at 680 °C for 4 h and crystallized at 790 °C for (**a**) 0.5 h, (**b**) 1 h, (**c**) 2 h, and (**d**) 4 h, where (**e**), (**f**), (**g**), and (**h**) are the high‑magnification (150x) three‑dimensional images of the regions in (**a**), (**b**) (**c**), and (**d**), respectively.


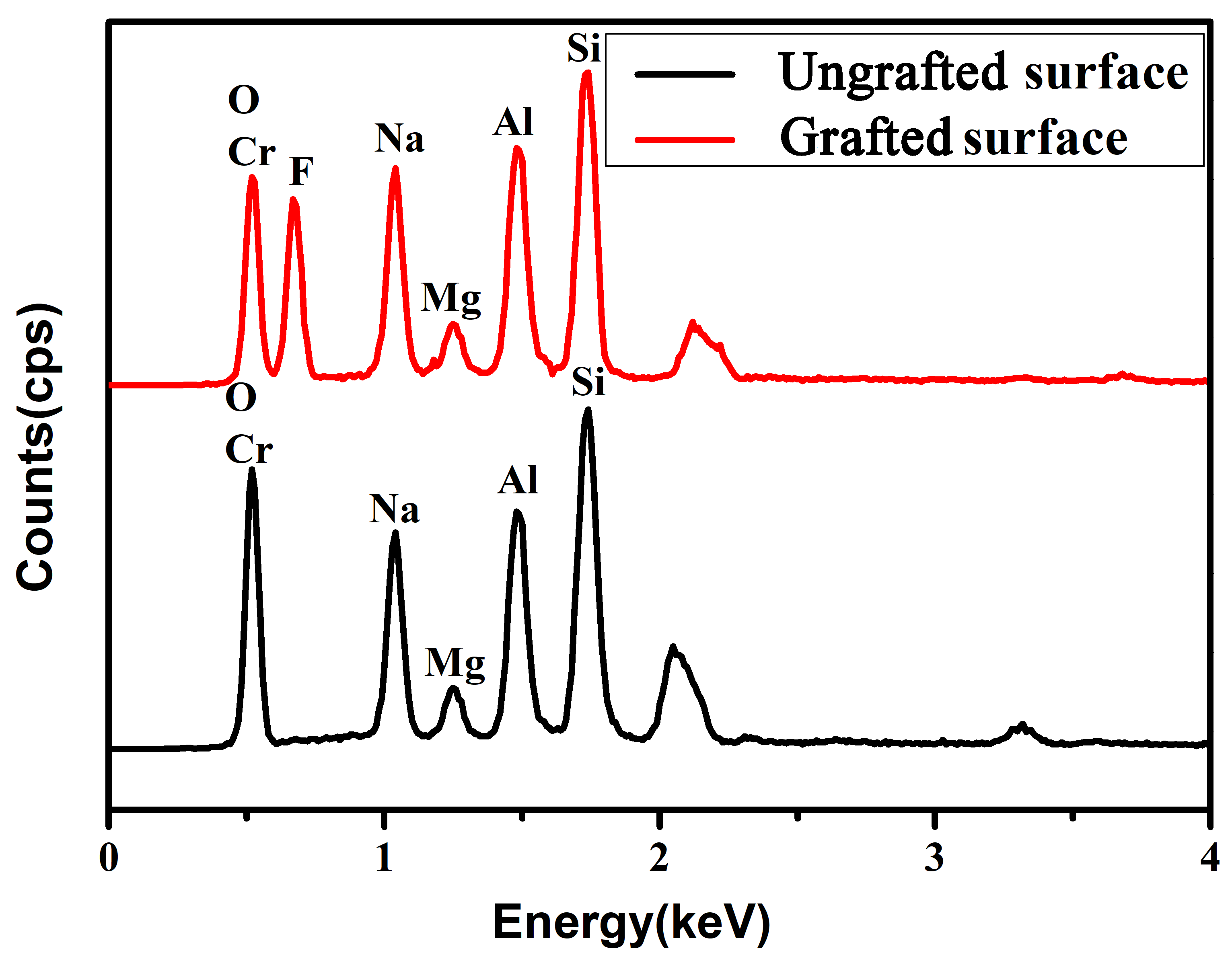


**Figure S4.** EDS patterns of the ungrafted and FAS‑17‑grafted glass‑ceramic surfaces.
